# Sex differences in the severity of presbyopia with dry eye: A cross-sectional retrospective study

**DOI:** 10.1371/journal.pone.0334117

**Published:** 2025-11-14

**Authors:** Masahiko Ayaki, Akiko Hanyuda, Kazuno Negishi

**Affiliations:** Department of Ophthalmology, Keio University School of Medicine, Tokyo, Japan; Saarland University, GERMANY

## Abstract

**Purpose:**

Visual symptoms in older people may derive from presbyopia and dry eye (DE) with sex-specific pathology. Previous studies have suggested women may have a greater risk for presbyopia than men of the same age. However, the association between DE, which is more frequent in women, and presbyopia has not been determined. This study explored whether the relationship between DE and the severity of presbyopia differs by sex.

**Methods:**

This cross-sectional retrospective cohort study included 1147 bilateral phakic patients, aged from 40 to 55 years (858 women and 289 men). Refraction, near add power at 30 cm, and DE-related clinical parameters (corneal staining score and tear break-up time [BUT]) were compared between the sexes. Correlation analyses and odds ratio comparisons of risk factors for reaching specific near add power (1.00, 1.25, 1.50, and 1.75 D) were performed.

**Results:**

Corneal staining and tear break-up time (BUT) were worse in women. Correlation analysis stratified by sex revealed that near add power correlated with age (women: β = 0.80, *P* < 0.01; men: β = 0.80, *P* < 0.01), and astigmatic errors (women: β = 0.16, *P* < 0.01; men: β = 0.19, *P* < 0.01) in both sexes, BUT only in women (β = −0.10, *P* < 0.01), and corneal staining only in men (β = 0.20, *P* < 0.01). Women with short BUT and men with corneal staining were more likely to reach higher near add power.

**Conclusion:**

The current study suggests that men with corneal staining and women with short BUT may need more near add power. These DE-related clinical features are significant factors for presbyopia and should be managed to ameliorate presbyopia-related symptoms.

## Introduction

Presbyopia is a disorder of the eye’s accommodation system characterized by a loss of the lens’s ability to focus on near objects [1,2]. Near vision has become increasingly critical in the recently digitalized society with portable and desktop displays [3]. Indeed, presbyopia is an emerging social and health issue with considerable economic impacts [4,5], especially for older people who may suffer from focusing difficulty. Presbyopia may be affected by numerous factors [2], including aging, the female sex [6], dry eye (DE) [7,8], and lifestyle [9,10]. Sex differences in presbyopia have been discussed in terms of arm-length and habitual near works [6]. Indeed, women seem to prefer under-corrected myopic contact lens power compared with men, suggesting that they prepare for near focusing difficulty, while men prefer to see far with full myopic correction [4]. Sex differences related to presbyopia have also been described for subjective happiness, sleep quality, and starting time for the first pair of reading glasses [9]. Women are more familiar with near tasks than men and see more closely than men due to shorter arm length and physical stature [11]. As such, sex differences in presbyopia have been suggested, but it is unknown what ocular surface factors contribute to it in medical settings. A meta-analysis has shown that women may have a greater preponderance for presbyopic eyeglasses with more add power than men of the same age [6], although there are no significant sex differences in accommodative amplitudes. These differences may be due to differences in preferred viewing distances or due to uncorrected hyperopia, and so the need for near-vision correction due to presbyopia depends, not just on the loss of focusing ability, but also on the habitual reading distance and the depth of focus [6]. To the best of our knowledge, ocular surface factors for sex differences in presbyopia have not been objectively identified.

DE is more frequent in women and it could be hypothesized that DE is a risk factor for presbyopia since sex has been suggested as a factor in both DE and presbyopia. Indeed, decreased visual function is a significant clinical issue in DE according to the criteria of the DE consensus report of the Asia Dry Eye Society [12]. Previous studies have shown that DE causes visual disturbances, including irregular astigmatism, higher order aberration, light scattering, and decreased functional vision [13–17], and that these symptoms can be improved by medication and other interventions, including mucin secretagogue eyedrops and punctal plug [18–20].

The aim of this study was to investigate the association of DE-related parameters on near add power. We focused on sex difference, which may affect the progression of presbyopia [21–24], especially in middle-aged DE patients as suggested previously [7].

## Methods

### Study design and participants

This study was a clinic-based, retrospective, cross-sectional cohort study involving healthy individuals attending the Otake Eye Clinic. The Institutional Review Board and Ethics Committee of the Kanagawa Medical Association (approval date, 12 November 2018; permission number krec2059006) approved the study, and it was carried out in accordance with the Declaration of Helsinki. The need for consent was waived by the Institutional Review Board. This study was a retrospective chart review for consecutive patients visiting from December 1, 2018 to December 31, 2022 for the first time, and patient interview and ocular examinations for analysis were routinely performed in the participating eye clinics. The Institutional Review Board and Ethics Committee of Keio University School of Medicine also approved this study (approval date, 28 June 2021; approval number 20210080) to permit authorship for authors (KN, AH and MA) who were appointed at the Keio University School of Medicine. The protocol was registered with the UMIN Clinical Trials Registry (UMIN000051891) on August 15, 2023. All the data collected in this study, including the patient interviews, were collected as part of routine standard-of-care examinations. Authors had access to information that could identify individual participants during or after data collection.

### Inclusion and exclusion criteria

Participants aged 40–55 years with bilateral phakic eyes and best-corrected visual acuity above 20/30 were included. According to a previous study [4], the amplitude of accommodation is strongly correlated with age between the ages of 40–55 years, indicating that this age range is suitable for analysis of the severity of presbyopia to explore risk factors other than age. Individuals were excluded if they used contact lenses, anti-glaucoma eye drops, or had severe cataract, vitreoretinal disease, any ocular surgery in the previous six months, or acute ocular disease in the previous two weeks.

### Ophthalmological examinations

Ophthalmological evaluation of participants consisted of best-corrected visual acuity (Vision Chart, SSC-370^R^; Nidek Co., Ltd., Gamagori, Aichi, Japan), autorefractometry (TonorefTM II; Nidek Co., Ltd.), slit-lamp biomicroscopy, and funduscopy. Binocular near add power was measured at a distance of 30 cm using a Bankoku near-acuity chart (Handaya Inc., Tokyo, Japan) [9]. After determining the patient’s distance refractive correction, the minimal additional power required to achieve near acuity above 20/25 at 30 cm was measured in 0.25 D increments, and was recorded as near add power.

DE-related examinations consisted of the tear break-up time (BUT) and the corneal staining test. BUT is a key diagnostic metric used to evaluate the stability of the tear film on the ocular surface. BUT was measured using a fluorescein filter paper strip (Ayumi Pharmaceutical, Tokyo, Japan), wetted with saline with the excess flicked off, applied at the lower lid margin. BUT was defined as the time interval between the third blink and the appearance of the first dark spot on the cornea. This was calculated from the mean of three measurements. Patients with a tear BUT ≤ 5 s are classified as having short BUT-type DE and it is a major diagnostic criteria of DE [12]. Corneal staining was used to detect corneal epitheliopathy, which often presents as multiple small epithelial defects in the corneal surface involving the corneal epithelial layer. Corneal staining scores were obtained by grading the intensity of staining observed one min after ocular administration of fluorescein dye. The National Eye Institute/Industry Workshop classification system was used, employing a yellow barrier filter, the slit lamp’s cobalt blue illumination, and a grading scale of 0–2 for severity and area. The clinical presentation of corneal epitheliopathy is generally called superficial punctate keratopathy (SPK) and it is a common corneal epithelial disorder characterized by scattered, pinpoint epithelial cell loss on the surface of the cornea. Its presence can signal underlying pathology and significantly affect ocular comfort and visual function.

### Statistical analysis

Patient demographics and ophthalmological parameters were presented as the median (Q1, Q3) for continuous variables and as percentages for categorical variables since age (*P* < 0.01; skewness −0.13, kurtosis −1.02) and near add power (*P* < 0.01; skewness −0.24, kurtosis −0.60) were not normally distributed with the Kolmogorov-Smirnov test. *T* tests and chi-squared tests were used to compare these demographics according to sex, as appropriate. To explore possible ophthalmic parameters that were associated with near add power, Spearman correlation analyses were conducted. Using multiple regression analysis, we investigated the correlation between near add power and the following parameters: age, spherical equivalent, astigmatic errors, anisometropia, tear BUT, and corneal staining score. Consequently, we selected age, astigmatic errors, BUT, and positive corneal staining as explained variables. We then estimated the odds ratios (ORs) and 95% confidence intervals for the presence of presbyopia (characterized by several cut-off points of near add power [< 1.00, 1.25, 1.50, and 1.75 D]) in relation to each selected ophthalmic parameter, using logistic regression models.

To visualize the sex-specific relationship between age and near add power, scatter plots were used. In exploratory analyses, we further examined whether the sex-specific relationship between age and near add power differed with the presence of DE (i.e., short BUT or presence of corneal staining). Regression lines and slopes were computed by the least-squares method. The differences in slope (ratio of age and near add power) and x-intercept between regression lines were analyzed by *t* tests. We performed all analyses using StatFlex (Atech, Osaka, Japan), with *P* < 0.05 considered to indicate a significant difference.

## Results

**Fig 1:**
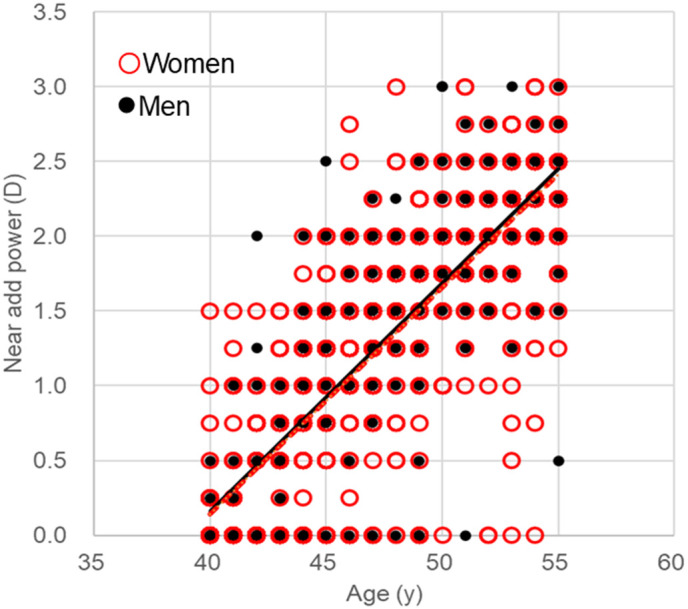
Scatter plots of near add power and age. Annual progression of near add power was similar in men (black circles, black solid regression line, y = 0.153x-5.96) and women (red circles, red dotted regression line, y = 0.152x-5.93; women vs. men, *P* = 0.861 for slope). Symbol overlap may not accurately represent the number of participants.

The present study included 1147 participants, of whom 858 were women (mean age 47.5 ± 4.5 years) and 289 were men (mean age 47.4 ± 4.4 years) (S1 Table). There were no significant differences in age, spherical equivalent, astigmatic errors, anisometropia, near add power, or proportion of participants at specific cut-off values of near add power between the sexes, whilst BUT (*P* < 0.01) and corneal staining (*P* < 0.01) were worse in women (Table 1). Scatter plots of near add power and age demonstrated the annual progression of near add power was similar in men and women (regression lines: y = 0.15x-5.96 and y = 0.15x-5.93, respectively; *P* = 0.86 for slope; Fig 1).

**Table 1:** Patient demographics and ophthalmological parameters.

	Total	Women	Men	*P*-value*
Number of cases	1147	858	289	
Age, y	47.5 (4.5)	47.5 (4.5)	47.4 (4.4)	0.66
Spherical equivalent, D	−3.73 (3.10)	−3.76 (3.06)	−3.64 (3.21)	0.58
Astigmatic errors, D	0.53 (0.59)	0.54 (0.59)	0.60 (0.71)	0.44
Anisometropia, D	0.56 (0.69)	0.54 (0.63)	0.63 (0.84)	0.08
Near add power, D	1.28 (0.86)	1.28 (0.86)	1.29 (0.86)	0.84
Tear break-up time^†^, s	3.8 (2.0)	3.5 (2.0)	4.6 (1.9)	< 0.01
Corneal staining^†^ (%)	22.9	26.4	12.8	< 0.01

Values are mean (standard deviation) unless indicated otherwise. *Women vs. men, unpaired *t*-test or chi-squared test, as appropriate. ^†^A detailed explanation of tear break-up time (BUT) and corneal staining can be found in the Methods section.

Correlation analysis stratified by sex revealed that near add power was associated with age (women: β = 0.80, *P* < 0.01; men: β = 0.80, *P* < 0.01) and astigmatic errors (women: β = 0.16, *P* < 0.01; men: β = 0.19, *P* < 0.01) in both sexes, and with short BUT in women only (β = −0.10, *P <* 0.01) and with corneal staining (SPK) in men only (β = 0.20, *P* < 0.01; Table 2). Multiple regression analysis indicated a significant correlation between near add power and age, as well as a weak association with corneal staining score in men (Table 3). Near add power showed significant correlations with age and astigmatic errors in women. Comparison of ORs for risk factors of reaching specific cut-off points of near add power indicated that women with a short BUT (near add power 1.50 and 1.75 D) and men with SPK (all near add power cut-off points tested) and with short BUT (1.25 D) were more likely to present with higher near add power (Table 4).

**Table 2:** Correlation between near add power and patient parameters.

Parameters	Women		Men	
	Linear regression β (SE)	Adjusted for age β (SE)	Linear regression β(SE)	Adjusted for age β (SE)
Age	0.79 (0.004)**		0.78 (0.004)**	
Spherical equivalent	0.02 (0.0001)	−0.01 (0.00006)	0.05 (0.0001)	0.02 (0.0001)
Astigmatic errors	0.14 (0.005)*	0.08 (0.0003)*	0.18 (0.0008)**	0.07 (0.0005)
Anisometropia	0.05 (0.0004)	0.05 (0.0003)*	0.05 (0.0005)	0.01 (0.0003)
Tear break-up time ^†^, s	−0.10 (0.01)**	−0.02 (0.01)	−0.12 (0.02)	−0.04 (0.01)
Corneal staining score ^†^	0.03 (0.04)	0.01 (0.03)	0.21 (0.10)**	0.09 (0.06)**

β=Standardized partial regression coefficient. *P < 0.05, **P < 0.01. SE, standard error. ^†^A detailed explanation of tear break-up time (BUT) and corneal staining can be found in the Methods section.

**Table 3:** Multiple regression analysis between near add power and patient parameters.

Parameters	Women	Men
Age	0.77 (0.004)**	0.74 (0.008)**
Spherical equivalent	0.01 (0.0007)	0.03 (0.0001)
Astigmatic errors	0.09 (0.003)**	0.06 (0.0004)
Anisometropia	0.03 (0.0003)	0.01 (0.0001)
Tear BUT	−0.02 (0.01)	−0.02 (0.1)
Corneal staining score	−0.02 (0.04)	0.07 (0.1)

Values are expressed as standardized partial regression coefficient (standard error). All parameters are included in the analysis. *P < 0.05, **P < 0.01. A detailed explanation of tear break-up time (BUT) and corneal staining can be found in the Methods section.

**Table 4:** Comparison of odds ratios for risk factors for developing symptomatic presbyopia^†^.

Characteristics	Women		Men	
	OR (95% CI)	*P*-value	OR (95% CI)	*P*-value
Age (continuous)	1.62 (1.51-1.74)	< 0.01	1.65 (1.48-1.84)	< 0.01
Spherical equivalent (continuous)	1.00 (1.00-1.01)	0.51	1.00 (0.99-1-00)	0.71
Astigmatic errors (continuous)	1.01 (1.00-1.01)	0.03	1.01 (1.00-1.01)	< 0.01
Anisometropia (continuous)	1.00 (0.99-1.00)	0.16	0.99 (0.99-1.00)	0.54
BUT (reference: BUT > 5)	1.84 (1.28-2.67)	< 0.01	1.15 (0.67-1.96)	0.59
Corneal staining (reference: no staining)	1.01 (0.77-1.54)	0.60	2.52 (1.23-5.17)	0.01

^†^Symptomatic presbyopia is defined in the Methods section.

BUT, tear break-up time; CI, confidence interval; OR, odds ratio.

**Fig 2:**
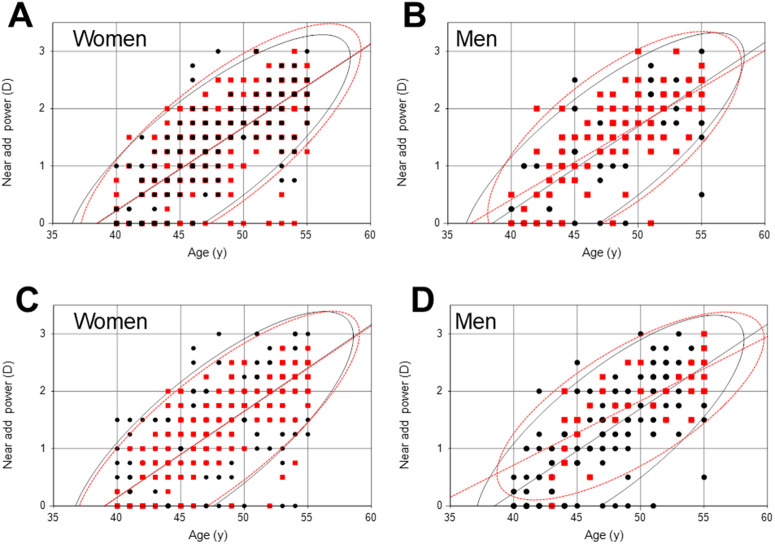
Scatter plots and regression lines with probability ellipses (95% confidence interval) showing the age-related distribution of near add power. Women (A) and men (B) with and without short tear break-up time (BUT). Red symbols, regression line, and probability ellipse show those with short BUT, while black symbols, line and ellipse show those without short BUT. The slopes of the regression lines for with and without short BUT were not different in women (regression line, y = 0.14x-5.58 and y = 0.14x-5.59, respectively; *P* = 0.99 for slope) or men (y = 0.13x-4.78 and y = 0.15x-5.96, respectively; *P* = 0.31 for slope). Women (C) and men (D) with and without superficial punctate keratopathy (SPK). Red symbols, regression line, and probability ellipse show those with SPK, while black symbols, line and ellipse show those without SPK. The distribution was similar in women with and without SPK, while men with SPK had higher near add power than those without SPK. The estimated x-intercept for with and without SPK was significantly different in men (34.2 and 39.1 years, respectively; *P* = 0.04) but not in women (39.3 and 38.8 years, respectively; *P* = 0.89). The slopes of the regression lines for with and without SPK were not different in men (regression line, y = 0.11x-3.76 and y = 0.15x-6.96, respectively; *P* = 0.06 for slope) or women (y = 0.15–5.83 and y = 0.15x-5.89, respectively; *P* = 0.88 for slope).

Scatter plots and regression lines of the age-related distribution of near add power with and without short BUT showed women with short BUT had higher near add power than women without short BUT, while there was no difference in men (Figs 2A, B). There was no difference in the slope of the regression lines between with and without short BUT in both women (*P* = 0.99) and men (*P* = 0.31). Scatter plots and regression lines of the age-related distribution of near add power with and without SPK showed a similar distribution in women with and without SPK, but men with SPK had higher near add power than those without SPK (Figs 2C, D). There was no difference in the slope of the regression line between with and without SPK in both women (*P* = 0.88) and men (*P* = 0.06). However, the estimated x-intercept was significantly different between with and without SPK in men (34.2 and 39.1 years, respectively; *P* = 0.04) but not in women (39.3 and 38.8 years, respectively; *P* = 0.89). The estimated near add power at the age of 45 was 1.19 D in men with SPK and 0.92 D in women with SPK.

## Discussion

The present study revealed women who have shorter BUTs and men with greater SPK are more likely to be symptomatically presbyopic [13–17]. However, the sex differences have not been fully elucidated, and the reason why women with short BUT and men with SPK are more likely to have higher near add power is unclear. Despite more blinking in women than men [25], higher-order aberration increases after blinking and the stability of higher-order aberration decreases in eyes with short BUT, and remains high in eyes with SPK [16]. Additionally, short BUT can induce glare sensitivity and fluctuating vision [16]. Kaido et al. [26] demonstrated microfluctuations of accommodation in individuals with short BUT-type DE. The age range of 40–55 years is the perimenopausal period for women when hormonal status drastically changes. Therefore, age may be a significant factor among female participants. However, short BUT can be ameliorated by treatment [19,20], and in this sense, presbyopia exacerbated by short BUT could be treatable.

Visual function may be worse in women than in men due to various factors, including sex hormones [27]. Women have more tear evaporation, higher tear osmolality, and a higher frequency of short BUT than men [28]. There is also speculation that men may have better discrimination in dynamic visual acuity than women [29]. In addition, the retina is thinner in women than men [30], supporting the theory that women’s visual function may be worse than men. Finally, estrogen deficiency worsens women’s vision by causing cataracts [31], and a recent investigation suggested ultraviolet A irradiation of the cornea induces greater DNA damage in female mice than male mice [32]. This sex-dependent effect of ultraviolet A is driven by the formation of reactive estrogen metabolites and estrogen-DNA adducts, leading to corneal epithelial and endothelial damage. Anthropologically, since the stone age, men needed good far vision for hunting, while women needed good near vision for agriculture and nursing [33]. Consequently, it could be speculated that the female visual system as a whole is adapted to near vision and the male visual system to far vision due to previous long-time lifestyles, but there is no evidence or study to support this theory.

Elderly men with SPK may suffer from impaired near vision earlier with higher near add power, compared with those without SPK due to increased corneal backward light scattering [16]. Men with SPK need more near add power than women, and, presumably, men are less susceptible to ocular surface disturbance since the OR for clinically significant presbyopia is representative of the burden of SPK in men; that is, the OR for SPK was greater than the OR for short BUT, suggesting that SPK was more harmful to men than short BUT. Indeed, the estimated near add power at the age of 45 was higher in men than in women with SPK. The precorneal tear film is repeatedly disrupted and smoothed with every blink, indicating short BUT could be overcome by blinking. However, SPK does not decrease simply by blinking. Additionally, DE-associated SPK remains unchanged for days or even years, while BUT can change anytime with blinking. These ocular surface conditions may contribute to the sex differences.

Ocular surface damage results from a wide variety of interacting factors, such as increased tear evaporation, hyperosmolarity, proinflammatory mediators in the tears, and decreased lubrication between the lids and globe of the eyes. Elderly men may also be troubled by a higher incidence of meibomian gland dysfunction (MGD) compared with women. MGD (defined as obstruction of meibomian orifices, absence of gland structure, or both) produces higher staining with fluorescein and rose bengal, compared with patients without MGD [34]. Androgens also have negative effects on meibomian gland epithelial cells and MGD worsens corneal aberrations and is improved with treatment [35]. Androgen deficiency may also lead to an increased incidence of cataracts [36].

The correlation between astigmatic error and near add power is debatable since a small astigmatic error is beneficial for depth of focus [37]. However, it is unclear whether it can be applied to the age group of 40–55 years in the present study, and the present results are consistent with previous reports describing no beneficial effects of astigmatism on near visual acuity [38].

We analyzed patients in the age range of 40–55 years which has been conventionally used in previous studies [21–23]. Near add power is a composite parameter and it should be assessed at age ranges when the relationship between age and near add power is roughly linear [21–24]. The age range of a previous study [7] was from 40 to 69 years and this range may have been too wide to explore the factor of presbyopia progression since an investigation of the relationship between accommodation amplitude and age [2] has suggested the status of presbyopia reaches a plateau around the age of 55, and it is well known that many people begin to feel difficulty in focusing near objects at around the age of 40 [1,2]. Therefore, we set the age range from 40 to 55 years as it would be relevant to evaluate the contributory factor to near add power when the amplitude of accommodation is rapidly decreasing in a roughly linear fashion [4].

The current study has several limitations. First, the lack of MGD findings and the lack of measurements of accommodation amplitude, corneal aberration, and pupillary diameter are a considerable limitation that should be addressed in subsequent studies. Second, this clinic-based study may not accurately represent the general population and there is an unbalanced gender ratio which may have limited statistical power to identify a gender difference since consecutive patients at first visit were reviewed to avoid selection bias. Third, additional information about physical and mental health, as well as family history, may also help explain the association between DE and presbyopia. DE itself is a multifactorial disease with many possible confounding factors, and it may potentially be affected by seasonal variations, systemic and psychiatric comorbidities, medications, and ocular complications. Furthermore, a detailed questionnaire on lifestyle factors, including smoking, drinking, exercise, diabetes and hypertension, would be necessary to comprehensively evaluate the interaction of lifestyle and presbyopia. Finally, the present results should be interpreted cautiously since near add power and age were not normally distributed.

In conclusion, the current study clarified DE is a significant factor for near add power by analyzing a cohort with ages ranging from 40 to 55 years when presbyopia rapidly progresses. The obtained results suggested that men with SPK and women with short BUT may need more near add power than those without. DE could be treated and such approach may help patients with DE suffering from presbyopia.

## Supporting information

S1 Table The raw data of the subjects. (XLSX)

## Abbreviations

DE: dry eye
OR: odds ratio
SPK: superficial punctate keratopathy
BUT: tear break-up time
